# TGFβ1 Induces Axonal Outgrowth via ALK5/PKA/SMURF1-Mediated Degradation of RhoA and Stabilization of PAR6

**DOI:** 10.1523/ENEURO.0104-20.2020

**Published:** 2020-09-28

**Authors:** Julia Kaiser, Martina Maibach, Ester Piovesana, Iris Salpeter, Nora Escher, Yannick Ormen, Martin E. Schwab

**Affiliations:** 1 Brain Research Institute, University of Zurich, CH-8057 Zurich, Switzerland; 2 Department of Health Sciences and Technology, ETH Zurich, CH-8057 Zurich, Switzerland

**Keywords:** downstream signaling, neurite outgrowth, plasticity, stroke, tgfb1

## Abstract

Transforming growth factor (TGF)β1 has repeatedly been associated with axonal regeneration and recovery after injury to the CNS. We found TGFβ1 upregulated in the stroke-denervated mouse spinal cord after ischemic injury to the motor cortex as early as 4 d postinjury (dpi) and persisting up to 28 dpi. Given the potential role of TGFβ1 in structural plasticity and functional recovery after stroke highlighted in several published studies, we investigated its downstream signaling in an *in vitro* model of neurite outgrowth. We found that in this model, TGFβ1 rescues neurite outgrowth under growth inhibitory conditions via the canonical TGFβR2/ALK5 signaling axis. Thereby, protein kinase A (PKA)-mediated phosphorylation of the E3 ubiquitin ligase SMURF1 induces a switch of its substrate preference from PAR6 to the Ras homolog A (RhoA), in this way enhancing outgrowth on the level of the cytoskeleton. This proposed mechanism of TGFβ1 signaling could underly the observed increase in structural plasticity after stroke *in vivo* as suggested by the temporal and spatial expression of TGFβ1. In accordance with previous publications, this study corroborates the potential of TGFβ1 and associated signaling cascades as a target for future therapeutic interventions to enhance structural plasticity and functional recovery for stroke patients.

## Visual Abstract

**Figure:**
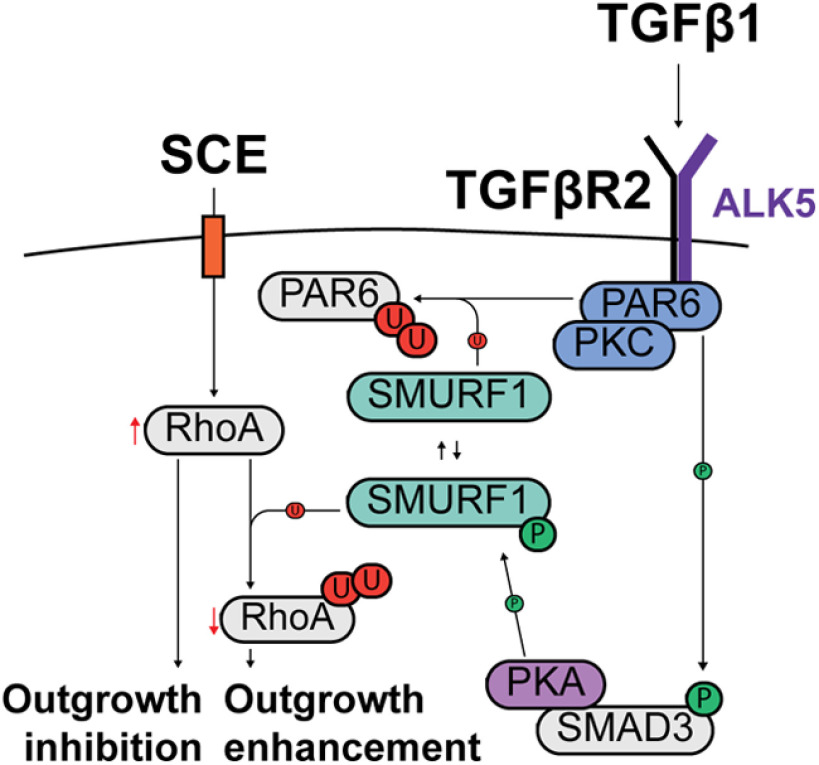

## Significance Statement

This study addresses a mechanism for Transforming growth factor (TGF)β1 to increase compensatory axonal sprouting and growth after cortical stroke, e.g., in the stroke-denervated cervical spinal cord, where it was previously implicated as a potential growth-inducer. The signaling pathway includes the canonical receptor components ALK5 and SMAD3 and a downstream modulation of the cytoskeleton via protein kinase A (PKA)/SMURF1 induced downregulation of Ras homolog A (RhoA) and upregulation of PAR6. Defining the downstream signaling pathway through which TGFβ1 can induce neurite outgrowth may provide new clinical targets for future therapeutic interventions to increase compensatory sprouting, thus contributing to functional recovery.

## Introduction

Interruption of the brain’s blood supply, e.g., in case of ischemic stroke, can result in life-long disability because of the loss of neurons (Murphy and Corbett, 2009; Cramer, 2018). Most available therapeutics for stroke patients target the acute phase in the hope to protect neurons from the ischemic damage, while rehabilitation to date remains the only treatment option for chronic stroke patients (Krakauer et al., 2012; Zeiler and Krakauer, 2013; Wahl and Schwab, 2014). Both in human stroke patients as well as animal models of stroke, the spontaneous recovery observed in the weeks after the incident has been attributed in parts to structural plasticity of healthy cortical neurons and connections, e.g., horizontal neurons in the peri-infarct region or the contralateral corticospinal neurons (CSNs) in case of large strokes (Carmichael et al., 2017). In this regard, a better understanding of the molecular cues inducing, guiding and maintaining this compensatory sprouting and rewiring response is pivotal to develop novel therapeutic approaches to enhance recovery after stroke. Inflammation-derived cytokines and locally released growth factors may have a beneficial effect on the repair mechanisms by directly promoting axonal regeneration (Benowitz and Popovich, 2011; Vidal et al., 2013). In the cortex surrounding a focal stroke lesion the upregulated cytokine growth differentiation factor 10 (GDF10) was reported to enhance structural plasticity and motor recovery (Li et al., 2015). GDF10 is a member of the highly evolutionarily conserved transforming growth factor (TGF) superfamily, which also includes the TGFβ and bone morphogenic protein (BMP) cytokine families (Weiss and Attisano, 2013; Zhang et al., 2017). A recent study highlighted the involvement of TGFβ1 signaling in neurogenesis and axonal regeneration in the peri-infarct cortex by viral knockdown of the co-receptor ALK5, leading to decreased levels of the growth associated protein GAP43 within the first two weeks after stroke, concomitant with a decrease in functional recovery (Zhang et al., 2019). A recently published transcriptomic screen showed an upregulation of TGFβ1 in the stroke-denervated cervical hemicord of adult mice at 28 d after a large cortical stroke. This finding suggests that TGFβ1 could be a mediator of the observed compensatory sprouting of the intact-side corticospinal tract (CST) and axon elongation/arborization in the spinal cord (Kaiser et al., 2019).

However, the mechanisms through which TGFβ1 may increase neurite outgrowth are still unclear. TGFβ1 binding to its receptor TGFβR2 can result in the formation of heteromeric complexes with two different type 1 receptors, ALK1 or ALK5, activating distinct downstream pathways often with opposing functions (Goumans et al., 2003; König et al., 2005; Stegmüller et al., 2008; Zou et al., 2009; Parikh et al., 2011; Finelli et al., 2013; Saijilafu et al., 2013; Hannila et al., 2013; Curado et al., 2014; Li et al., 2015). TGFβ1 promotes axonal outgrowth *in vitro* in primary neurons, including cortical neurons (Abe et al., 1996; Knöferle et al., 2010; Li et al., 2015) and blockage of the receptor TGFβR2 leads to shorter axons (Yi et al., 2010). Intriguingly, direct activation of SMAD2, the canonical downstream target of TGFβ1 signaling, reduced axonal outgrowth in some instances (Stegmüller et al., 2008; Knöferle et al., 2010). This discrepancy of axonal growth induction versus inhibition may stem from canonical versus non-canonical signaling of TGFβ1. A better understanding of the downstream signaling pathway through which TGFβ1 can induce axonal outgrowth is, therefore, urgently needed; it may also provide interesting new clinical targets for future therapeutic interventions.

In the present study, we show that TGFβ1 is transcriptionally upregulated in the region of the axotomized cervical CST and in the premotor layers of the CST-deprived spinal gray matter as early as 4 d postinjury (dpi). Using a simple but highly reproducible *in vitro* assay, we demonstrate that TGFβ1 treatment rescues neurite outgrowth in the growth inhibitory environment of crude spinal cord extract (SCE) through the canonical TGFβ1/ALK5 signaling axis. Further hypothesis driven pharmacological blockade studies suggest an underlying signaling mechanism involving a PKA-mediated phosphorylation of the E3 ubiquitin ligase SMURF1, switching its substrate preference from PAR6 to Ras homolog A (RhoA). In conclusion, we show that besides canonical transcriptional changes associated with TGFβ1, it also enhances neurite outgrowth by downregulating RhoA, the downstream signaling mediator of many CNS-associated growth inhibitory molecules. Thereby TGFβ1 has the potential to prime the neuronal cytoskeleton into a growth permissive state despite of the inhibitory CNS environment.

## Materials and Methods

### Animals

All animal experiments were performed with the approval of and in strict accordance with the guidelines of the Zurich Cantonal Veterinary Office. A total of *n *=* *21 adult C57BL/6J mice (two to three months, 20–28 g, female, Charles River Laboratories) were used in this study. Only one sex was used to minimize lesion size variability and animal numbers. Animals were housed in groups of four to five under a constant 12/12 h light/dark cycle with food and water *ad libitum*.

### Photothrombotic stroke

For all surgeries, mice were initially anesthetized using 3–4% isoflurane, transferred to a stereotactic frame (Kopf Instruments) and kept at 1–2% isoflurane throughout the surgery. Body temperature was maintained at 37°C on a heating pad. All animals received a unilateral photothrombotic stroke to lesion the right side sensorimotor cortex as previously described (Watson et al., 1985; Kaiser et al., 2019). Briefly, the skull was exposed by a midline incision of the scalp. An opaque template with a defined opening (3 × 5 mm) was aligned to the midline over the right motor and premotor cortex (−2 to +3 mm A/P, 0–3 mm M/L related to bregma). Five minutes after intraperitoneal injection of 0.1 ml Rose Bengal (10 mg/ml in 0.9% NaCl; Sigma-Aldrich), the skull was illuminated for 10.5 min with a cold light source (Olympus, KL1500LDC, 150 W, 3000 K) placed firmly on top of the skull. Control animals were given a sham operation without illumination of the skull. Postoperative care included recovery on a heating mat, sustained analgesia provided via drinking water (Novalgin, 2 mg/ml with 5% sucrose) and antibiotic treatment where necessary for 3 d.

### Behavioral testing

Behavioral tests were performed before surgery (baseline) as well as 4, 7, 14, and 28 d after photothrombotic stroke (dpi) of the right motor cortex. Poststroke impairment and recovery of forelimb function was assessed using the cylinder test. Forelimb paw touches to the cylinder wall during spontaneous rearing behavior were recorded for 20 min or 30 rears in total (*n *=* *12). Paw dragging of the affected limb was scored as the percentage of paw drags divided by total number of paw touches (Roome and Vanderluit, 2015). The horizontal ladder walk test was used as an additional, more sensitive assessment of skilled limb placement (Metz and Whishaw, 2009). Mice were habituated to the ladder run 1 d before test recordings; no further training was conducted. Three trials on a 40-cm-long ladder with irregularly spaced rungs of 1- to 2-cm distance were recorded on each testing day (*n *=* *12). The number of foot errors was measured as the number of total misplacements (slips) divided by the total number of steps taken. All recorded steps were analyzed and no videos were excluded for the analyses to avoid bias toward stroked groups (*n *=* *3 videos per animal per test day).

### Perfusion fixation and tissue processing

All mice were terminally anesthetized with 3–5% isoflurane followed by injection of pentobarbital (300 mg/kg body weight, i.p.; Streuli Pharma AG). Animals were transcardially perfused with ice-cold Ringer’s solution [containing 10^5^ IU/l heparin (Roche) and 0.25% NaNO_2_]. Brains and spinal cords were quickly dissected and snap-frozen on dry ice to preserve RNA quality. The brains were immersed in 4% PFA overnight before being transferred to 30% sucrose in phosphate buffer (PB) for cryoprotection. Samples were blinded from the point of tissue harvesting.

### Analysis of lesion completeness

For the accurate analysis of lesion size, brain cross-sections (40 μm) were stained on-slide with cresyl violet solution for 1 min, dehydrated in a series of increasing ethanol concentrations and washed in Xylol before coverslipping with Eukitt (Sigma-Aldrich). Brain sections at four defined landmarks (1.98, 0.98, −0.22, and −1.34 mm, in relation to bregma) were analyzed for stroke volume and depth of the cortical lesion. Average cortical stroke depth was calculated as the average of lesion depth across these four landmarks.

### *In situ* hybridization

Sections (16 μm) of fresh frozen tissue were cut on a cryostat at −20°C and stored at −80°C. *In situ* gene expression was assessed using the RNAScope protocol (Advanced Cell Diagnostics, RNAscope Fluorescent Multiplex Assay) according to the manufacturer’s protocol. Briefly, slices were fixed in 4% PFA for 20 min before being hybridized to the probe (RNAScope: TGF-β1, catalog #407751), which was further amplified using the branched DNA amplification methods. Sections were counterstained with DAPI and digitalized (Zeiss, Axio Scan.Z1, 200×). For analysis using ImageJ/FIJI, only slices with tears or folds were excluded; *n* = 3 sections per animal per group and spinal levels C5 and C6 were randomly selected. Quantification of mRNA expression was expressed as the percentage of white signal over black background in defined regions (CST in the dorsal funiculus and intermediate gray matter (iGM) of the denervated hemicord) after applying a threshold that was manually defined for five randomly selected images of the dataset and averaged.

### Neurite outgrowth assay

Candidate factors were tested using a previously described neurite outgrowth assay (Maibach et al., 2020). Briefly, N1E-115 mouse neuroblastoma cells were plated at a density of 10,000 cells/cm^2^ in Neurobasal medium supplemented with 2% L-glutamine and 1% PenStrep to induce neuron-like differentiation. After 24 h of differentiation the cells were supplemented with crude adult rat spinal cord CHAPS extract, and candidate factors or pharmacological agents were added (Maibach et al., 2020). The following proteins and molecules were used at the indicated concentrations: 1 ng/ml TGFβ1 (R&D Systems), 1 nm TEW7197 (Selleckchem, S7530), 1 nm ML347 (Selleckchem, S7148), 10 nm SIS3 (Selleckchem, S7959), 10 nm A01 (Merck, SML1404), 10 nm Ht31 (Tocris Biotechne, 6286), and 10 nm ATM (Merck, 157201). After an additional 24-h outgrowth phase, the cells were fixed and counterstained with Coomassie solution (0.25% Coomassie Brilliant Blue R250, 50% MeOH, 10% HoAC). Images were acquired randomly over the wells at 10× magnification. Mean neurite outgrowth per cell was quantified in ImageJ by applying a grid to the pictures and counting intersections of neurites with the grid lines and relating this number to the total number of cell bodies in the corresponding well (Rønn et al., 2000). Experiments were conducted in five biological replicates with three technical replicates per condition.

### Immunoblotting

Cells were washed twice in PBS on ice and lysed in RIPA buffer (150 mm NaCl, 1% NP-40, 1% sodium deoxycholate, 0.1% SDS, and 50 mm Tris; pH 8) containing 2× HALT phosphatase inhibitor cocktail and 5 mm EDTA. The lysates were incubated on ice for 30 min and centrifuged at 13,000 × *g* for 15 min at 4°C. The supernatants were collected and stored at –80°C.

The samples were prepared in Laemmli buffer (Bio-Rad) supplemented with 10% βMEtOH and denatured at 90°C for 3 min. The samples were separated on pre-cast 4–15% Mini PROTEAN R TXG TM gels (Bio-Rad) at 250 V in Tris-glycine running buffer (25 mm Tris, 192 mm glycine, and 0.1% SDS; pH 8.3). Proteins were transferred onto a 0.45 μm PVDF membrane in Tris-glycine transfer buffer (25 mm Tris, 19 2 mm glycine, and 20% MeOH) for 90 min with a constant current of 300 mA. Subsequently, membranes were blocked for 1 h with 5% BSA (Sigma) in TBS-T (10 mm Tris, 150 mm NaCl, and 0.01% Tween 20; pH 7.5) and probed with primary antibodies overnight at 4°C. The membranes were washed three times in TBS-T, probed with secondary HRP-coupled antibodies (Thermo Fisher Scientific) at a concentration of 0.05–0.1 μg/ml for 1 h at room temperature (RT) and washed again three times in TBS-T. Detection was performed using SuperSignal West PICO (Thermo Scientific) or WesternBright Sirius (Advansta) chemiluminescent substrates, and images were acquired on the Gel Doc imager (Bio-Rad). Densitometry analysis was performed with ImageJ/FIJI software, and values were normalized to the housekeeping gene GAPDH or total protein and the mean value of the corresponding control group. For Western blotting, the primary antibodies are summarized in Table 1.

**Table 1:** Antibodies used for Western blotting

Target	Host species		Dilution	Clone	Company	Catalogue number
p-SMAD3 S423/425	Rabbit		1:1000	C25A9	Cell Signaling Technology	9520
SMAD2/3	Rabbit		1:1000	D7G7	Cell Signaling Technology	8685
RhoA	Rabbit		1:1000	67B9	Cell Signaling Technology	2117
PAR6	Rabbit		1:500		Abcam	ab111823
p-ERK1/2 T202/Y204	Rabbit		1:2000		Cell Signaling Technology	9101
ERK1/2	Rabbit		1:1000	137F5	Cell Signaling Technology	4695
p-S6P S235/236	Rabbit		1:1000	D57.2.2E	Cell Signaling Technology	4858
S6P	Mouse		1:1000	54D2	Cell Signaling Technology	2317
TGFβR2	Goat		1:500		R&D Systems	AF-241
GAPDH	Mouse		1:20,000	6C5	Abcam	ab8245

### RhoA assessment in growth cones

Cells were fixed for 15 min in 4% PFA and subsequently blocked and permeabilized in blocking buffer (0.1% Triton X-100 and 10% BSA in PBS) for 1 h. Primary antibody [mouse anti-RhoA (Santa Cruz, SC-418), 1:500] was applied in 1% BSA in PBS and incubated over night at 4°C. The samples were washed three times in PBS, followed by incubation with secondary antibodies (1 μg/ml anti-mouse Cy3-coupled antibodies; Invitrogen, A10521, A10520) in 1% BSA in PBS for 1 h at RT. After washing three times in PBS, cells were counterstained with 1:100 Phalloidin Alexa Fluor 488 (Invitrogen, A12379) to visualize the actin cytoskeleton and DAPI (50 nm, Invitrogen, D3571) as a nuclear counterstain. The samples were coverslipped and imaged at 200×. For image acquisition, exposure times were kept constant and below gray scale saturation. For immunofluorescence normalization in ImageJ/FIJI, the signal in the phalloidin channel was thresholded and an area mask was created around the fluorescent object. This mask was then applied onto the RhoA pictures and the total pixel intensity within the area was measured. This value was normalized to the area of the growth cone or cell body. Similarly, a nuclear counter stain was used to define the area of the nucleus.

### Statistical analysis

Statistical analysis was performed with Prism 7.0 (GraphPad Software). To detect differences between groups and within groups over time, two-way ANOVA followed by Dunnett’s multiple-comparisons (MC) was used. Other symbols might be used to indicate two comparisons in one graph. In bar graphs, all data are plotted as mean standard error of the mean (±SEM), while data were normalized to solvent controls in the neurite outgrowth assays. Dots in the behavioral graphs represent individual animals. For all neurite outgrowth assays, *n* = 5 biological replicates with *n* = 3 technical replicates per condition. 200–400 cells per technical replicate were counted. Throughout the manuscript, *p ≤* *0.05, **p ≤* *0.01, and ***p ≤* *0.001.

## Results

### TGFβ1 is endogenously upregulated at early time points in the stroke denervated hemicord

**Figure 1.**
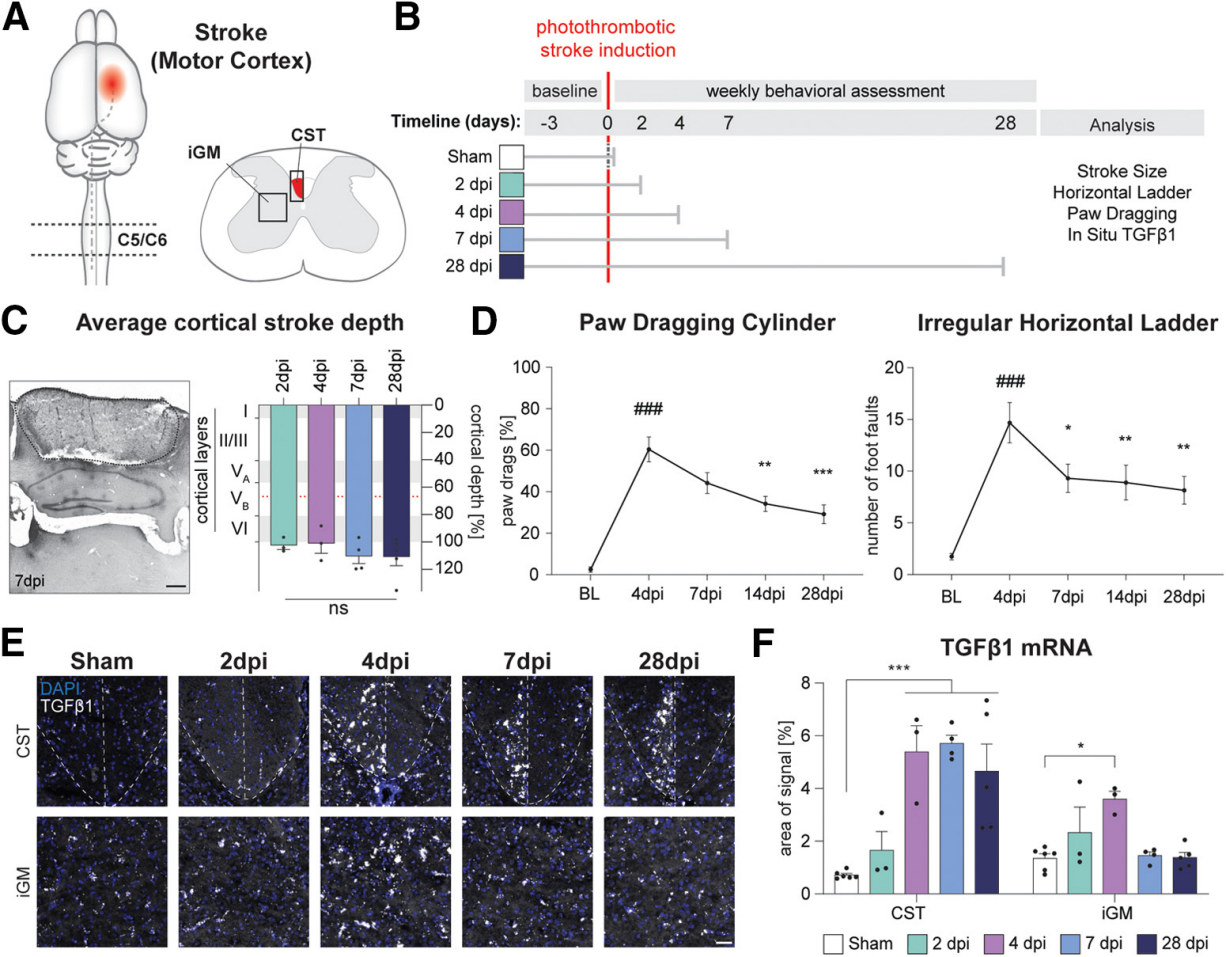
TGFβ1 is upregulated in the stroke-denervated cervical spinal cord at early time points, transiently in the iGM and persistently in the degenerating CST in the dorsal funiculus. ***A***, ***B***, Experimental study timeline: adult female C57BL/6 mice received a large unilateral cortical stroke (sham: *n* = 6; 2 dpi: *n* = 3; 4 dpi: *n* = 3; 7 dpi: *n* = 4; 28 dpi: *n* = 5; total: *n* = 21). All animals were tested behaviorally and euthanized at 2, 4, 7, or 28 dpi, respectively. ***C***, Average cortical stroke depth showed no significant differences among the experimental groups with strokes reaching into deep cortical layers to the corpus callosum (100%). Estimated location of CSNs in Layer V is indicated by a red dotted line. The sensorimotor area was specifically injured (Nissl staining at 7 dpi, representative picture). Scale bar = 500 μm. ***D***, Behavioral analysis for paw drags in the cylinder test and number of missteps on the irregular horizontal ladder shows that stroke induction resulted in a deficit in motor behavior with a subsequent, partial functional recovery within 28 dpi. # is used for statistical comparison to baseline levels, * for comparison to 4 dpi. ***E***, Representative pictures of TGFβ1 mRNA expression in the degenerating CST and iGM of the spinal levels C5/6 in sham animals as well as in mice at 2, 4, 7, and 28 dpi. Scale bar = 50 μm. ***F***, Quantification of the percentage of area signal in the CST shows an upregulation of TGFβ1 mRNA starting at 4 dpi and persisting until 28 dpi. In the iGM, TGFβ1 mRNA is transiently upregulated at 2 and 4 dpi. *p <* *0.05, **p <* *0.01, ***p <* *0.001.

In mice, a significant degree of spontaneous re-innervation of the stroke denervated cervical spinal cord by sprouting of contralesional CSNs can be detected 28 d after large lesions to the motor cortex (Ueno et al., 2012; Bachmann et al., 2014; Kaiser et al., 2019). At this late time point after stroke, TGFβ1 expression was found to be upregulated in the cortex (Lehrmann et al., 1995; Knuckey et al., 1996; Ata et al., 1999; Zhu et al., 2001; Doyle et al., 2010) as well as in the stroke-denervated hemicord (Kaiser et al., 2019). However, sprouting of contralateral cortical axons may be initiated much earlier in the stroke-denervated hemicord. To address whether TGFβ1 could serve as a trigger and modulator of structural plasticity in the stroke-denervated hemicord, we evaluated the expression pattern of TGFβ1 within the dorsal funiculus and gray matter at selected, early time points after stroke (2, 4, 7, and 28 dpi; Fig. 1*A*,*B*). We ensured that stroke lesions were consistent in size and location at all time points. Strokes were primarily localized to the sensory-motor cortex including the premotor regions and successfully ablated all cortical layers with little to no impact on the corpus callosum or deeper structures (Fig. 1*C*) .This stroke model induced a behavioral deficit in the forelimbs which recovered partially over the course of four weeks as assessed by paw dragging and foot faults in the horizontal ladder task (Fig. 1*D*). *In situ* hybridization for TGFβ1 in the stroke-denervated spinal cord showed an increase of TGFβ1 mRNA at 4 dpi in both analyzed regions, the CST domain of the dorsal funiculus as well as the iGM (Fig. 1*E*,*F*). This increased expression is transient in the iGM and persists up to 28 dpi in the CST, suggesting that TGFβ1 is present at time points when growth initiation and axon elongation and arborization of contralesional CSNs and other tracts occur.

### TGFβ1 rescues neurite outgrowth in inhibitory environment via canonical ALK5/SMAD3 signaling

**Figure 2.**
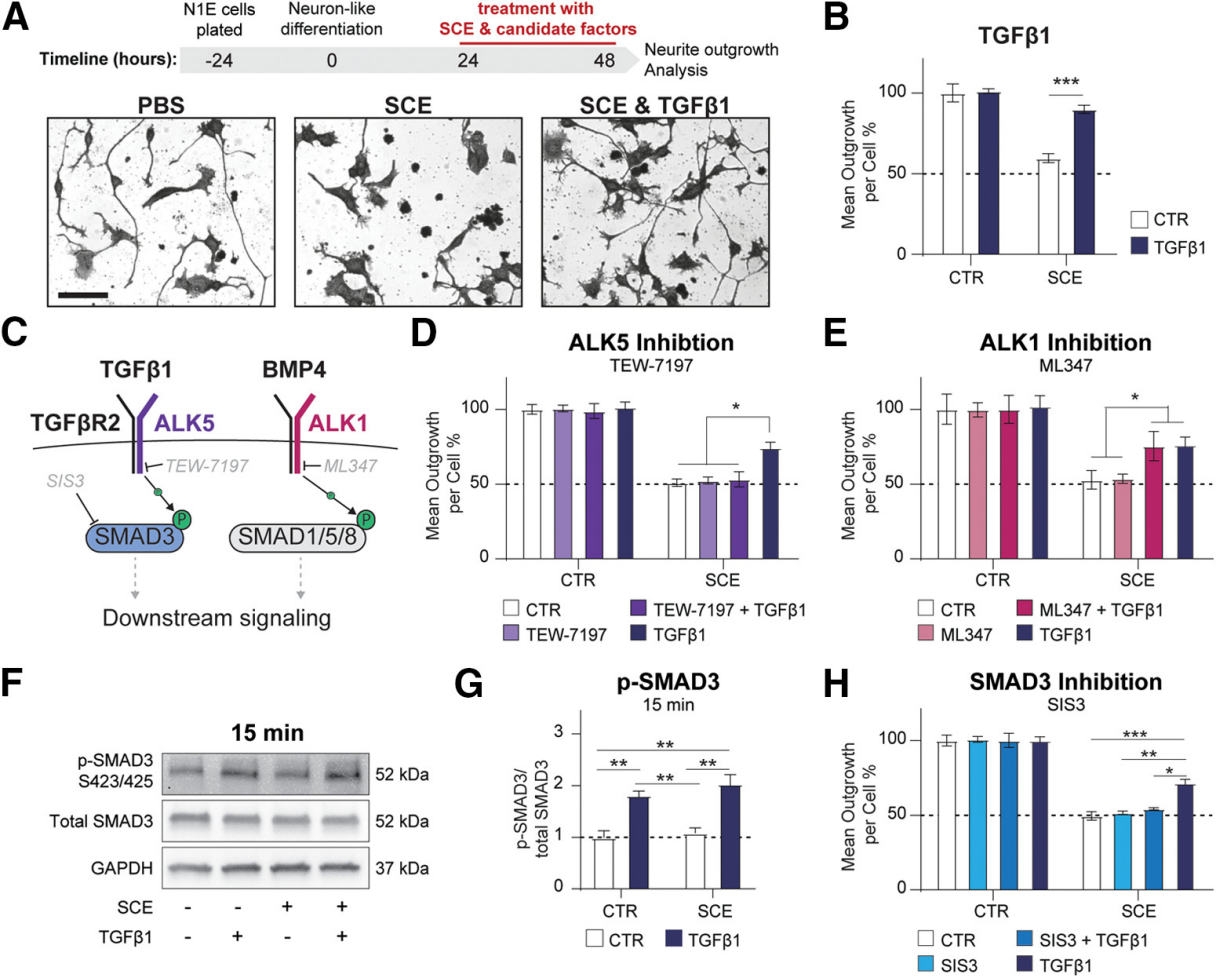
TGFβ1 rescues neurite outgrowth in inhibitory environment via canonical TGFβR2/ALK5/SMAD3 signaling axis. ***A***, Timeline for neurite outgrowth assay: N1E-115 neuron-like cells were plated and differentiated for 24 h by serum deprivation. Candidate factors with/without SCE were supplemented for a 24-h outgrowth period, after which neurite outgrowth was assessed. Neurite outgrowth from N1E-115 cells (left panel) is inhibited by SCE (middle panel) and rescued by SCE in combination with TGFβ1 (right panel). Scale bar = 50 μm. ***B***, Quantification of mean outgrowth per cell in presence or absence of SCE or 1 ng/ml TGFβ1 normalized to solvent control condition. ***C***, Schematic representation of the pathways of TGFβ1 signaling axis. ***D***, Quantification of mean outgrowth per cell of SCE-treated N1E-115 cells in presence of either ALK5 inhibitor (TEW-7197) or TGFβ1 or combined treatment. ***E***, Quantification of mean outgrowth per cell of SCE-treated N1E-115 cells in presence of either ALK1 inhibitor (ML348) or TGFβ1 or combined treatment. ***F***, ***G***, Representative Western blottings for p-SMAD3, total SMAD3 or GAPDH of cells treated with SCE and TGFβ1 or both in combination after 15 min (***F***) and quantification thereof (***G***). ***H***, Quantification of mean outgrowth per cell of SCE-treated N1E-115 cells with either SMAD3 inhibitor (SIS3) or TGFβ1 or combined treatment. Means ± SEM of five independent experiments are shown; each experiment has three wells with 200–400 cells per well per condition; *p <* *0.05, **p <* *0.01, ***p <* *0.001.

We first investigated the potential of TGFβ1 to rescue neurite outgrowth in a growth inhibitory environment. To model the inhibitory *in vivo* CNS environment, we used our previously established neurite outgrowth assay (Maibach et al., 2020). In this assay, application of crude spinal cord CHAPS extract (SCE) to the differentiated neuron-like cell line N1E-115 over a 24-h process formation phase, inhibits outgrowth in a dose-dependent manner, without affecting cell survival. As TGFβ1 was shown to increase axon length in neurons, including primary cortical neurons (Abe et al., 1996; Knöferle et al., 2010; Li et al., 2015), we first established an effect of TGFβ1 in our neurite outgrowth model. We found that already nano molar concentrations of TGFβ1 were able to restore neurite outgrowth of IC50 SCE-treated N1E-115 cells to ∼80% (Fig. 2*A*,*B*).

We investigated the specific receptor complex through which the neurite outgrowth promoting TGFβ1 signaling might be transduced (Fig. 2*C*). While pharmacological inhibition of ALK1 had no effect on the TGFβ1-mediated rescue of neurite outgrowth in the inhibitory SCE treatment conditions, inhibition of ALK5 prevented the TGFβ1-mediated rescue (Fig. 2*D*,*E*). This suggests that the TGFβ1 signal is propagated via the canonical TGFβR2/ALK5 signaling receptor complex. Phospho-profiling of the ALK5 downstream effector SMAD3 revealed an increased phosphorylation/activation of SMAD3 in the TGFβ1-treated conditions (Fig. 2*F*,*G*). Accordingly, pharmacological inhibition of SMAD3 prevented the TGFβ1-mediated rescue effect (Fig. 2*H*). Taken together, these results demonstrate that TGFβ1 signals via the canonical TGFβR2/ALK5/SMAD3 axis to rescue neurite outgrowth under CNS growth inhibitory conditions.

### TGFβ1 induces SMURF1-mediated downregulation of RhoA and stabilization of PAR6

We did not observe an increase in neurite outgrowth in the absence of SCE after 24 h of TGFβ1 treatment alone (Fig. 1*B*) with the extremely low TGFβ1 concentration used in this study. This lack of a general growth promoting effect suggests that TGFβ1 signaling specifically interferes with and cancels the SCE-induced growth inhibitory signaling cascades. We therefore hypothesized that TGFβ1 could negatively influence the RhoA/Rho-associated, coiled-coil containing protein kinase (ROCK) pathway, a key downstream signaling effector of many growth inhibitory molecules (Thiede-Stan and Schwab, 2015). One possible mechanism for such crosstalk could be the SMURF1-mediated RhoA ubiquitination which targets RhoA for degradation and thereby enhances neurite outgrowth (Wang et al., 2003; Smith et al., 2005; Sahai et al., 2007; Vohra et al., 2007; Narimatsu et al., 2009). The substrate preference of SMURF1 is modulated by PKA-dependent phosphorylation, which increases its binding affinity for RhoA relative to PAR6 (Cheng et al., 2011). In parallel to enhanced RhoA degradation, the membrane associated PAR6/PKC complex is stabilized. Both PKA activation (via association with SMAD3) and induction of a PAR6/protein kinase C (PKC) complex at the membrane have been reported as downstream effectors of TGFβ1 signaling in endothelial cells (Ozdamar et al., 2005; Wang et al., 1998; Yang et al., 2013), making it a possible mechanism for the observed rescue of neurite outgrowth by TGFβ1.

**Figure 3.**
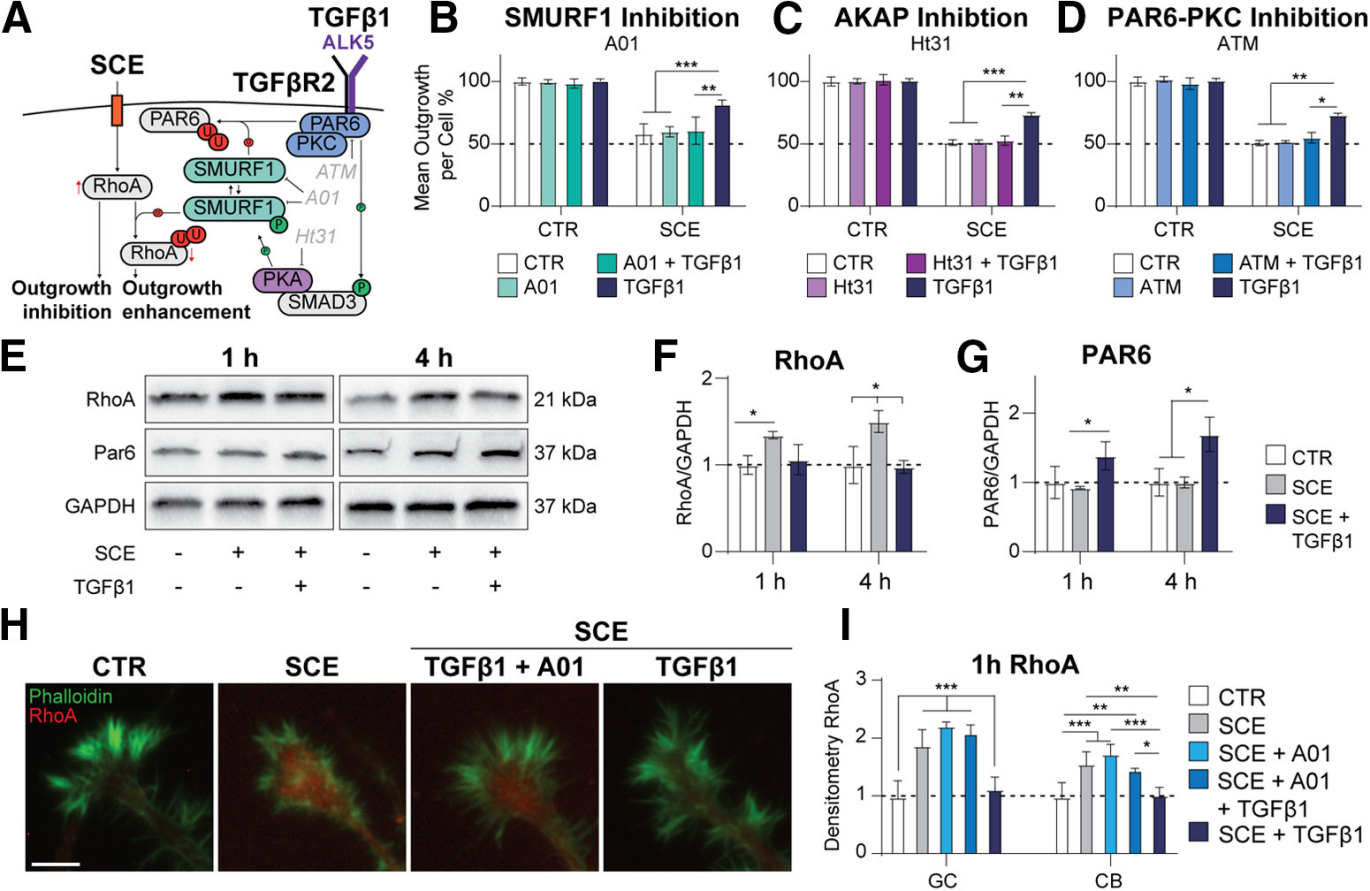
TGFβ1 induces SMURF1-mediated downregulation of RhoA. ***A***, Schematic representation of the downstream signaling pathway proposed in this study whereby activated SMAD3 activates PKA which turns P-SMURF1 to RhoA for ubiquitination; in turn, the PAR6/PKC complex is stabilized. ***B***, Quantification of mean outgrowth per cell of SCE-treated N1E-115 cells with either SMURF1 inhibitor (A01) or TGFβ1 or combined treatment. ***C***, Quantification of mean outgrowth per cell of SCE-treated N1E-115 cells with either AKAP inhibitor (Ht31) or TGFβ1 or combined treatment. ***D***, Quantification of mean outgrowth per cell of SCE-treated N1E-115 cells with either PAR6-PKC binding inhibitor (ATM) or TGFβ1 or combined treatment. ***E–G***, Representative Western blottings for RhoA and PAR6 in N1E-115 cells ± SCE and/or TGFβ1 (***E***) and quantification thereof (***F***, ***G***). ***H***, Representative pictures of growth cones stained for RhoA (red) and f-actin cytoskeleton (green). Scale bar = 10 μm. ***I***, Quantification of RhoA in growth cones (GC) and cell bodies (CB), respectively; *p <* *0.05, **p <* *0.01, ***p <* *0.001.

To test this hypothesis, we inhibited selected key molecules of the proposed non-canonical signaling cascade (Fig. 3*A*). Inhibition of SMURF1 by the blocker A01 (Cao et al., 2014) prevented the TGFβ1 elicited rescue of neurite outgrowth from SCE- induced growth inhibition (Fig. 3*B*). Likewise, inhibition of substrate recruiting PKA scaffolding protein A kinase anchoring protein (AKAP) by Ht31 (Kennedy and Scott, 2015) as well as pharmacological inhibition of PKC and PAR6 interaction ATM (Butler et al., 2015; Erdogan et al., 2006; Stallings-Mann et al., 2006) resulted in the loss of the TGFβ1-mediated rescue effect (Fig. 3*C*,*D*). Furthermore, TGFβ1 co-treatment with SCE resulted in a significant downregulation of RhoA levels both globally (Fig. 3*E*,*F*) and locally in growth cones (Fig. 3*H*,*I*). This reduction of RhoA was associated with increased PAR6 levels (Fig. 3*E*,*G*), supporting the hypothesis of a TGFβ1 induced switch in SMURF1 substrate preference leading to lower levels of RhoA and higher levels of PAR6 as an underlying mechanism for the observed neurite outgrowth recovery.

## Discussion

We observed an upregulation of TGFβ1 mRNA at 4 dpi in the stroke-affected CST and the iGM (laminae 5–7) in the cervical spinal cord after a large stroke to the motor cortex, a time point when compensatory axonal sprouting of the CST may be triggered. This increased expression persisted in the CST area in the dorsal funiculus up to 28 dpi. *In vitro* neurite outgrowth analysis showed that TGFβ1 can rescue neurite outgrowth through the canonical ALK5/SMAD3 signaling axis in a growth inhibitory environment. Downstream of the TGFβR2/ALK5 receptor, TGFβ1 led to the accumulation of PAR6, and negatively impacted on the RhoA/ROCK pathway by reducing the SCE-induced accumulation of RhoA. We propose an underlying signaling mechanism involving PKA-dependent SMURF1 regulation, leading to a substrate preference switch of SMURF1 from PAR6 to RhoA, thereby decreasing RhoA and increasing the growth enhancing state of the cytoskeleton. Such a mechanism could be the basis on which TGFβ1 mediates compensatory sprouting of re-innervating CSN axons in the stroke-denervated spinal cord.

Following stroke, TGFβ1 has been reported to exert neuroprotective effects. Administration of TGFβ1 led to reduced infarct sizes (Gross et al., 1993; Prehn et al., 1993), while blocking TGFβ1 signaling led to increased ischemic damage (Ruocco et al., 1999). Direct neuronal or glial effects could not be distinguished from anti-inflammatory effects, however. Evidence for TGFβ1 as a regulator of axonal growth is sparse and contradictory. Several *in vitro* studies correlated TGFβ1 treatment with axonal growth promotion in several subtypes of neurons (Abe et al., 1996; Stegmüller et al., 2008; Knöferle et al., 2010; Walshe et al., 2011; Li et al., 2015), while others reported an inhibitory or no effect at all (Ho et al., 2000; Do et al., 2013; Jaskova et al., 2014). These varying effects may be driven by context-specific and cell-specific signaling of TGFβ (Massagué, 2012); our present finding that TGFβ1 alone did not affect neurite outgrowth, but reversed the growth inhibitory effect of a crude spinal cord protein extract are in line with such a concept. The importance of studying the effects of TGFβ1 on axonal growth *in vivo* is thus evident, however, interpretation of the results may be complex owing to the pleiotropic roles of TGFβ in the control of cell proliferation, differentiation, wound healing and immune system (Kang et al., 2009; Morikawa et al., 2016).

Previous studies have reported indirect effects of TGFβ1 on modulation of axonal growth (Li et al., 2017), e.g., by induction of astrocyte proliferation (Kohta et al., 2009). Endogenous TGFβ1 may also serve as a trophic factor as TGFβ1 deficiency leads to an exacerbated neuronal cell death after facial nerve axotomy (Makwana et al., 2007). TGFβ1 can be secreted by astrocytes and by immune cells, thereby often functioning as an immunosuppressant (Vidal et al., 2013; Liddelow et al., 2017; Tripathi et al., 2017). Recently, TGFβ1/ALK5 signaling was associated with increased dendritic plasticity of cortical neurons, axonal sprouting of corticorubral projections and increased motor recovery after ischemic reperfusion injury (Zhang et al., 2019).

A previous study reported an upregulation of TGFβ1 mRNA in the stroke-denervated spinal cord at 28 dpi (Kaiser et al., 2019), suggesting that TGFβ1 might serve as a local tissue-derived pro-regenerative cue for re-innervating axons after cortical stroke. Here, we observed an increase in TGFβ1 mRNA as early as 4 dpi. While we found a transient increased expression of TGFβ1 in the iGM, this increase persisted in the dorsal funiculus in the CST area. Interestingly, this temporal and spatial expression profile matches the previously described activation of microglia and infiltration of macrophages (Kaiser et al., 2019), suggesting that these cells may be the predominant source of TGFβ1 in the stroke-denervated spinal cord. This interpretation is in line with a previous finding of increased TGFβ1 expression in microglia/macrophages in the stroke penumbra early after middle cerebral artery occlusion (Doyle et al., 2010).

We addressed the potential downstream pathways of TGFβ1 leading to increased neurite outgrowth under growth inhibitory conditions. We conducted our studies using aa *in vitro* model the neuroblastoma-derived cell line N1E-115 treated with crude SCE (Maibach et al., 2020). While this model lacks the direct translational impact of primary cell cultures, it ensures a high reproducibility between studies that primary cultures fail to provide owing to an often large batch-to-batch variability and contamination with non-neuronal cells. High sensitivity and reproducibility are a crucial prerequisite for a routine assay that is used for therapeutic compound screening. By using the well characterized neuron-like N1E cells, we were also able to avoid non-neuronal cell contamination by, e.g., astrocytes, which might serve as a primary source for TGFβ1 and thus interfere with our results in this study. An additional advantage of this assay is the ability to distinguish effects of the treatment on neurite outgrowth directly versus neuronal cell death as counted by number of cells. Treatment of N1E cells with SCE did not lead to an increase in cell death, but to a stunted neurite outgrowth with cells portraying a “panning” like phenotype. Co-treatment with TGFβ1 led to a robust increase in neurite outgrowth under these inhibitory conditions. Moreover, the neurite growth inhibitory effects of SCE in the N1E cultures were highly comparable to those previously published in primary cortical or hippocampal neurons, and the TGFβ1-mediated rescue of neurite outgrowth from SCE-induced growth inhibition in our *in vitro* model of N1E-115 cells was dependent on ALK5 and SMAD3, as was shown to be the case for cortical neurons *in vivo* (Zhang et al., 2019).

We then went on to pharmacologically block selected downstream targets of TGFβ1/ALK5 signaling to identify potential effects on neurite outgrowth in this model. By applying the candidate factors in a well-defined time frame after initial neurite outgrowth and in combination with inhibitory SCE, we were able to address post-translational signaling mechanisms, including phosphorylation and ubiquitination. While these studies allow us to address the direct effects of pharmacological interventions, we did not address effects on a transcriptional level in this study.

Application of SCE was shown to increase RhoA levels both in the cell body as well as in the growth cone. RhoA activity is associated with neurite retraction in response to growth cone collapsing agents (Feltrin and Pertz, 2012; Jeon et al., 2012). Pharmacological blockade of RhoA or the downstream effector Rho-kinase (ROCK) abolished the inhibitory effects of SCE, specifically also of its active ingredients Nogo-A and MAG (Niederöst et al., 2002). TGFβ1 treatment was associated with a downregulation of RhoA back to baseline levels (control group without SCE treatment), indicating that TGFβ1 signaling might have led to a decreased response to the inhibitory molecules of SCE such as Nogo-A or MAG.

We further saw an accumulation of PAR6 protein in the TGFβ1-treated N1E-115 cells, suggesting increased protein stability or synthesis resulting from the TGFβ1 treatment. Previous studies have described TGFβ1-mediated regulation of proteasomal processes in neuronal cells (Tadlock et al., 2003; Knöferle et al., 2010). Along these lines, we found that the TGFβ1-mediated rescue of neurite outgrowth depended on the E3 ubiquitin ligase SMURF1 as well as PKC/PAR6 interaction. In various cell types, TGFβ1 signaling induces cellular polarity by recruitment and activation of the PAR6/PKC complex to TGFβR2 (Ozdamar et al., 2005). Other studies suggested that this complex relocates SMURF1 to the membrane, where it induces RhoA degradation (Wang et al., 2003; Sahai et al., 2007; Narimatsu et al., 2009) and neurite outgrowth (Bryan et al., 2005; Vohra et al., 2007).

Notably, in our study, the TGFβ1 induced neurite outgrowth was also dependent on PKA activation. Previous studies have shown that PKA is a downstream target of TGFβ1 and is associated with SMAD proteins (Wang et al., 1998; Yang et al., 2013). PKA-mediated phosphorylation of the ubiquitin ligase SMURF1 in the RhoA interaction domain was shown to increase SMURF1 affinity for RhoA relative to PAR6, thereby contributing to axon formation *in vitro* and neuronal polarization *in vivo* (Cheng et al., 2011). It is possible that TGFβ1 signaling may also influence additional Rho-like GTPases such as Rac and Cdc42 and effectively regulate neurite outgrowth through additional signaling pathways (Zhang, 2009). Based on the present data, we propose that TGFβ1 enhances axonal outgrowth via stabilization of PAR6 and degradation of RhoA via a PKA/SMURF1-dependent mechanism. These results can guide future experiments to analyze these mechanisms *in vivo* and in particular in CSNs after a cortical stroke.

Endogenous TGFβ1 levels were shown to be increased in early phases after stroke, within the critical period of plasticity (Doyle et al., 2010). While the present and other studies suggest that prolonged treatment with TGFβ1 or a downstream effector of the TGFβ1/ALK5 signaling pathway may be used to enhance plasticity and extend its critical window after stroke (Li et al., 2015; Zhang et al., 2019), a systemic delivery of TGFβ1 would bring about unwanted negative side-effects owing to the plethora of functions of TGFβ1 as a inflammation related molecule (Akhurst and Hata, 2012). It is thus necessary to understand the specific signaling mechanisms by which TGFβ1 mediates structural plasticity and axonal outgrowth, both *in vitro* and *in vivo*, to develop a successful therapeutic agent.

The present study investigates a potential signaling mechanism through which TGFβ1 induces neurite outgrowth in a growth inhibitory environment *in vitro* and possibly compensatory axonal sprouting *in vivo*. This signaling pathway includes the canonical receptor components ALK5 and SMAD3 and a downstream activation of the cytoskeleton via PKA/SMURF1 induced downregulation of RhoA and upregulation of PAR6.

## Synthesis

Reviewing Editor: Lynda Erskine, University of Aberdeen Decisions are customarily a result of the Reviewing Editor and the peer reviewers coming together and discussing their recommendations until a consensus is reached. When revisions are invited, a fact-based synthesis statement explaining their decision and outlining what is needed to prepare a revision will be listed below.

The authors have thoroughly addressed the comments in the previous reviews. It provides a novel mechanism involving TGFB1 in compensatory axonal sprouting and growth after stroke.

## Author Response

Dear Dr. Erskine,

many thanks for the very helpful comments and suggestions by you and the reviewers. We hereby submit our revised manuscript in accordance with the suggested changes, additions and adaptations. As this study is based on a previously published study that highlights TGF1 in the context of stroke and justifies the hypothesis that we were following in this manuscript, we would like to ask if we may proceed with a non-double-blind review process.

Please find attached the manuscript with changes highlighted in red, a point-by-point response to the concerns of the reviewers (see below) as well as the adapted figures.

With friendly regards

Julia Kaiser, Martina Maibach and Martin E. Schwab

-

Point-to-point response to the concerns of the reviewers:

Major points

1. The authors may overstate the conclusions that can be drawn from their work, especially declaring a role for TGF1 in compensatory axonal sprouting post-stroke. The only in vivo evidence described within is TGF1 expression in the spinal cord post-stroke.

We adapted the manuscript to further define the proposed role of TGF1. While we found that TGF1 is expressed in the stroke-denervated hemicord as early as 4 days post injury, the manuscript goes on to explore one potential downstream signaling mechanism through which TGF1 may mediate neurite outgrowth in an in vitro model. It remains to be seen whether this is one of the mechanisms through which TGF1 mediates post-stroke compensatory sprouting of corticospinal axons. However, several additional studies indicate an influence of TGF1 on dendritic/axonal sprouting of cortical neurons (Abe et al., 1996; Knöferle et al., 2010; Li et al., 2015; Zhang et al., 2019).

2. The neuroblastoma cell culture model is not a clinically relevant in vitro model to assess neurite outgrowth. Primary neuronal cultures, such as postnatal rat cortical neuron cultures, should be used instead. This is particularly relevant to the CST that the authors focused on. Key experiments for Figure 3 should be replicated using primary cortical neuron cultures.

We conducted our studies using a previously established in vitro model of neuroblastoma-derived cell line N1E-115 treated with crude spinal cord extract (SCE) (Maibach et al., 2020). While this model lacks the direct translational impact of primary cell cultures, it ensures a high reproducibility between studies that primary cultures fail to provide owing to an often large batch-to-batch variability and contamination with non-neuronal cells. High sensitivity and reproducibility is a crucial prerequisite for a routine assay that is used for therapeutic compound screening. By using the well characterized neuron-like N1E cells, we were also able to avoid non-neuronal cell contamination by e.g. astrocytes, which might serve as a primary source for TGF1 and thus interfere with our results in this study. Moreover, the neurite growth inhibitory effects of SCE in the N1E cultures were highly comparable to those previously published in primary cortical or hippocampal neurons, and the TGF1 mediated rescue of neurite outgrowth from SCE induced growth inhibition in our in vitro model of N1E-115 cells was dependent on ALK5 and SMAD3, as was shown to be the case for cortical neurons in vivo (Zhang et al., 2019).

This paragraph was also added to the manuscript (p. 14-15)

3. The authors discussed throughout the manuscript that TGF1 can induce neurite outgrowth and therefore is a promising strategy to increase compensatory sprouting of contralateral CST axons. However, the evidence produced here with the neuroblastoma cell culture model showed that TGF1 alone had no effects on neurite outgrowth (Fig2B). The effect of TGF1 on neurite outgrowth following SCE treatment is likely via neuroprotective mechanism by TGF1. It is obvious from Fig2A, the cells under SCE treatment alone were having “sick” appearance, suggesting cell death. The authors need to carry out experiments to differentiate between the claimed “growth promoting” effect and possible “neuroprotective” effect.

Our previously established in vitro model to address neurite outgrowth was added as an additional reference to better describe the model. An additional advantage of this assay is the ability to distinguish effects of the treatment on neurite outgrowth directly vs. neuronal cell death as counted by number of cells. Treatment of N1E cells with SCE did not lead to an increase in cell death, but to a stunted neurite outgrowth with cells portraying a “panning” like phenotype. Instead, co-treatment with low concentrations of TGF1 (much lower than in the previous studies showing an effect on primary cortical neurons (Li et al., 2015)) led to a robust increase in neurite outgrowth under these inhibitory conditions.

4. The authors hypothesize the lack of increased neurite growth in the presence of TGF1 alone is due to TGF1 negatively influencing RhoA activity. Could it be that the lack of effect is due to the cells being primed to grow just by nature of being replated, i.e. they are in an enhanced growth state as it is?

In the described experiments of this manuscripts, cells were not replated. N1E-115 mouse neuroblastoma cells were plated at a density of 10’000 cells/cm2 in Neurobasal medium supplemented with 2% L-glutamine and 1% PenStrep to induce neuron-like differentiation. After 24h of differentiation the cells were supplemented with crude adult rat spinal cord CHAPS extract, and candidate factors or pharmacological agents were added for an additional 24h (Maibach et al., 2020). This was rephrased in the Materials & methods to better describe the experimental timeline. We further added a schematic timeline to figure 2.

5. The authors used solely pharmacological approach in experiments for Fig2 and Fig3 trying to establish the link of molecules in the TGF1-ALK5-PKA-SMURF1- RhoA pathway. However, this approach alone is not sufficient and convincing. Alternative genetic approach such as using siRNA to knockdown SMAD3, PKA, and SMURF1 should be used to replicate some of the key findings with the pharmacological approach.

In this manuscript, we used pharmacological approaches to address post-translational mechanisms. The effects described are seen on a post-translational level including phosphorylation and ubiquitination, not on a transcriptional level. We would not expect a change in mRNA levels based on our pharmacological treatments. Additionally, a pharmacological approach brings the advantage that they can be applied in a very defined window of time and are reversible without evoking compensatory mechanisms. We added a discussion of the limitations and advantages of this approach to the manuscript (p. 15)

6. The authors demonstrate that application of SCE is sufficient to increase expression of RhoA. Is there any effect on outgrowth when SCE is coapplied with a RhoA inhibitor such as Rhosin?

We added this important discussion point to the manuscript:

Application of SCE was shown to increase RhoA levels both in the cell body as well as in the growth cone. RhoA activity is associated with neurite retraction in response to growth cone collapsing agents (Feltrin and Pertz, 2012; Jeon et al., 2012). Pharmacological blockade of RhoA or the downstream effector Rho-kinase (ROCK) abolished the inhibitory effects of SCE, specifically also of its active ingredients Nogo-A and MAG (Niederöst et al., 2002). TGF1 treatment was associated with a downregulation of RhoA back to baseline levels (control group without SCE treatment), indicating that TGF1 signaling might have led to a decreased response to the inhibitory molecules of SCE such as Nogo-A or MAG. (p. 15-16)

7. The authors provided no evidence to directly link SMAD3 and PKA or SMURF1. The authors should carry out qRT-PCR to assess mRNA expression levels of PKA or SMURF1 in cells treated with or without SMAD3 inhibitor SIS3. Similar experiments shall be conducted in cells treated with or without PKA inhibitor to assess mRNA levels of SMURF1.

The effects described are seen on a post-translational level including phosphorylation and ubiquitination, not on a transcriptional level. While we cannot exclude it, we would not expect a change in mRNA levels based on our pharmacological treatments in a qRT-PCR. We added this limitation as a discussion point to the manuscript (p. 15)

8. Is there any change in the levels of SMURF1 with SCE application? The authors demonstrate a clear pathway between SMURF1 activation and RhoA degradation, but it is interesting that inhibition of SMURF1 using A01 does not result in an increase in RhoA protein expression - is it possible application of SCE itself has an effect on SMURF1 expression?

According to our working hypothesis SMURF1 primarily targets PAR6 for degradation under inhibitory growth conditions modeled by the spinal cord extract. This substrate preference is switched to RhoA by TGF1 signaling. Using the present data set, we cannot make a statement whether PAR6 is the default target of SMURF1 or whether SMURF1 gets actively recruited to the growth cone and primed to tag PAR6 for degradation due to some inhibitory cues presented in the SCE. Coincidentally, SCE treatment induces an accumulation of RhoA, which is probably induced by a Nogo-A/MAPK mediated signaling (Maibach2020). Therefore, we would not expect that co-treatment of SCE with A01 mediated SMURF1 inhibition would significantly increase RhoA levels further. However, we do agree that the question of potential modulation of SMURF1 levels, location or activation pattern by the growth inhibitory cues of the SCE is intriguing in itself and would consider addressing this in a future line of experimentation that does not investigate the TGF1 downstream signaling in the context of neurite outgrowth.

9. The authors failed to exclude/discuss the possibility that TGF1 may promote outgrowth via PKA-CREB route, or directly impact on Cdc42/Rac which then inhibits Rho.

We added this important discussion point to the manuscript (p. 16). It is possible that TGF1 signaling may also influence additional Rho-like GTPases such as Rac and Cdc42 and effectively regulate neurite outgrowth through additional signaling pathways (Zhang, 2009). Based on the present data, we propose that TGF1 may enhance axonal outgrowth via stabilization of PAR6 and degradation of RhoA via a PKA/SMURF1 dependent mechanism but cannot exclude additional mechanisms.

10. Is there involvement of the white matter in the strokes performed? Representative images of the brain sections should be included in order to evaluate depth and width of the strokes.

In these studies, photothrombotic strokes were primarily localized to the cortical layers with little to no impact on the corpus callosum white matter parts (see analysis of cortical stroke depth, Fig 1C, where 100% represents cortical thickness). We added a representative picture to further demonstrate the extent of the cortical lesion, as well as references to the previous study in which the model was further described (Kaiser et al., 2019).

11. Can the authors hypothesize how long the beneficial effects of TGF1 application persist? Can a short-lived response explain why increased sprouting and plasticity is usually only seen in early time points post-stroke?

We have not addressed endogenous expression of TGF1 after 28dpi, however, TGF1 expression was elevated between 4dpi up to 28dpi in the dorsal funiculus after cortical stroke. In the same time frame we can also see ongoing compensatory sprouting of contralesional corticospinal neurons within the stroke-denervated hemicord after cortical stroke (Kaiser et al., 2019). However, systemic delivery of TGF1 might have additional side-effects that can be detrimental to recovery (Akhurst and Hata, 2012).

The following discussion point was added to the manuscript to address this concern:

Endogenous TGF1 levels were shown to be increased in early phases after stroke, within the critical period of plasticity (Doyle et al., 2010). While the present and other studies suggest that prolonged treatment with TGF1 or a downstream effector of the TGF1/ALK5 signaling pathway may be used to enhance plasticity and extend its critical window after stroke (Li et al., 2015; Zhang et al., 2019), a systemic delivery of TGF1 would bring about unwanted negative side-effects owing to the plethora of functions of TGF1 as a inflammation related molecule (Akhurst and Hata, 2012). It is thus necessary to understand the specific signaling mechanisms by which TGF1 mediates structural plasticity and axonal outgrowth, both in vitro and in vivo, to develop a successful therapeutic agent.

Minor points

1. Please provide a citation for the study referencing a transcriptomic screen of the mouse cervical hemicord (presumably Kaiser et al. 2019).

As this manuscript might benefit from a non-blinded editorial process, we added all references to the previous studies that indicated TGF1in the stroke-denervated hemicord to make it easier to follow this study.

2. There are some inconsistencies with gene names, e.g. Smurf1 vs SMURF1

We changed inconsistencies throughout the manuscript.

3. Why were only female mice used for the experiment?

No difference was found between males and females either in stroke size or in the total fiber density over the whole grey matter at spinal levels C5/C6 over any of the measured time points (Fig 1, measured as described in (Kaiser et al., 2019)). Thus, we decided to use female mice only for this study as it allowed us to perform stroke surgeries without having to adjust for animals’ weight by increasing/decreasing the illumination time, thereby decreasing stroke size variability and ultimately also reducing animal numbers.

Fig 1. Sex distribution in stroke size and total fiber density in the denervated hemicord at spinal level C5/C6 after cortical stroke. A) no difference was found between male and female C57BL/6J mice in average cortical stroke depth B) male and female mice do not show a difference in their total fiber density across the grey matter of the denervated hemicord within time point groups.

4. Evaluation of in situ hybridization should perhaps be normalized to number of cells present in each image - the authors do note that TGF1 can be secreted by invading macrophages.

We did not normalize TGF1 expression as measured by in situ hybridization to number of cells present as it is a secreted molecule and thus the amount of mRNA / cell does not reflect the amount of protein that may induce axonal sprouting of neurons. While it is clearly an important question for future research, the source of TGF1 in the stroke-denervated hemicord is not addressed in this manuscript as it might be released by any of the cells present in this environment (Doyle et al., 2010). Notably, the expression pattern of TGF1 matches both spatially and temporally the activation pattern observed in Iba1+ cells in our previous publication (Kaiser et al., 2019), however, astrocytes and neurons might also be involved in the release of TGF1 (Vidal et al., 2013; Cekanaviciute et al., 2014; Liddelow et al., 2017; Tripathi et al., 2017). This important point has been has been reformulated and incorporated into the discussion chapter (p. 14).

5. Representative images of RhoA staining in cell bodies should be included alongside growth cone representative images, or possibly whole cell images with a zoom in to a region of interest.

Subfigure 3 H - I should convince the reader that the observed changes in RhoA levels of the whole cell lysates analysis (Subfigure 3 E-F) is relevant for growth cone dynamics and outgrowth and not only due to large scale changes in the cell body. Due to the cells size, we imaged growth cones and cell bodies separately in each condition randomly and did not take lower magnification images to serve as overviews. Unfortunately, for these reasons, we are unable to supply the matching cell body to the portrayed growth cone. However, we do think that they do not add substantially to the readers conclusion as the focus of this manuscript is the outgrowth of neurites and the dynamics of RhoA at the growth cone tip after co-treatment with SCE and TGF1.

6. Page 2: last 4 sentences - there is a need of a reference.

The reference here was previously excluded due to the blinded editorial process. We added the reference to the sentence in question.

7. Throughout the manuscript, there is a major problem of describing/explaining experimental unit either in the in vivo and in vitro studies. For example, n=21 mice, but how many each group, e.g. sham, and stroke animals? For histology, how many animals per time point assessed? For in vitro work, how many biological replicates, and how many technical replicates per biological replicate? How many cells were analysed for neurite outgrowth?

We added the following to the materials & methods: For all neurite outgrowth assays, n=5 biological replicates with n=3 technical replicates per condition. 200-400 cells per technical replicate were counted. Throughout the manuscript, *=p {less than or equal to} 0.05, **=p {less than or equal to} 0.01, and ***=p {less than or equal to} 0.001.

8. Page 4: for horizontal ladder, there was no description of training conducted before tests.

We added the following to the materials & methods: Mice were habituated to the ladder run one day prior to test recordings; no further training was conducted.

9. For analysis of in situ hybridisation and RhoA immunostaining, no mentioning of software used.

All analysis was done in FIJI/ImageJ. References to the software was further added to the appropriate sections within materials & methods.

10. Page6: spinal cord extract - need to describe how this was achieved/produced, the optimisation data of spinal cord extract on neuroblastoma cell growth should be provided.

We have added references & descriptions to our previously established model of neurite outgrowth where we also describe the optimization methods to ensure successful inhibition of neurite outgrowth using the SCE (Maibach et al., 2020).

11. Page 8: statistical analysis needs clarification if there were non-parametric data, which is quite likely in histological findings.

We further defined the statistics both in the materials & methods part as well as in the figure legends.

References

Abe K, Chu PJ, Ishihara A, Saito H (1996) Transforming growth factor-β 1 promotes re-elongation of injured axons of cultured rat hippocampal neurons. Brain Res 723:206-209.

Akhurst RJ, Hata A (2012) Targeting the TGF signalling pathway in disease. 11.

Cekanaviciute E, Fathali N, Doyle KP, Williams AM, Han J, Buckwalter MS (2014) Astrocytic transforming growth factor-beta signaling reduces subacute neuroinflammation after stroke in mice. Glia 62:1227-1240.

Doyle KP, Cekanaviciute E, Mamer LE, Buckwalter MS (2010) TGF signaling in the brain increases with aging and signals to astrocytes and innate immune cells in the weeks after stroke. J Neuroinflammation 7:62.

Feltrin D, Pertz O (2012) Assessment of Rho GTPase signaling during neurite outgrowth. In: Rho GTPases, pp 181-194. Springer.

Jeon C-Y, Moon M-Y, Kim J-H, Kim H-J, Kim J-G, Li Y, Jin J-K, Kim P-H, Kim H-C, Meier KE, others (2012) Control of neurite outgrowth by RhoA inactivation. J Neurochem 120:684-698.

Kaiser J, Maibach M, Salpeter I, Hagenbuch N, Souza VBC, Robinson MD, Schwab ME (2019) The spinal transcriptome after cortical stroke - In search of molecular factors regulating spontaneous recovery in the spinal cord. J Neurosci 39:2571-18.

Knöferle J, Ramljak S, Koch JC, Tönges L, Asif AR, Michel U, Wouters FS, Heermann S, Krieglstein K, Zerr I, Bähr M, Lingor P (2010) TGF- 1 enhances neurite outgrowth via regulation of proteasome function and EFABP. Neurobiol Dis 38:395-404.

Li S, Nie EH, Yin Y, Benowitz LI, Tung S, Vinters H V, Bahjat FR, Stenzel-Poore MP, Kawaguchi R, Coppola G, Carmichael ST (2015) GDF10 is a signal for axonal sprouting and functional recovery after stroke. Nat Neurosci 18:1737-1745.

Liddelow SA et al. (2017) Neurotoxic reactive astrocytes are induced by activated microglia. Nature.

Maibach MA, Piovesana E, Kaiser J, Holm MM, Risic Z, Maurer M, Schwab ME (2020) Nogo-A Beyond the RhoA/ROCK Pathway - Novel Components of Intracellular Nogo-A Signaling Cascades. bioRxiv:2020.07.10.197368 Available at: http://biorxiv.org/content/early/2020/07/11/2020.07.10.197368.abstract.

Niederöst B, Oertle T, Fritsche J, McKinney RA, Bandtlow CE (2002) Nogo-A and myelin-associated glycoprotein mediate neurite growth inhibition by antagonistic regulation of RhoA and Rac1. J Neurosci 22:10368-10376.

Tripathi P, Rodriguez-Muela N, Klim JR, de Boer AS, Agrawal S, Sandoe J, Lopes CS, Ogliari KS, Williams LA, Shear M, Rubin LL, Eggan K, Zhou Q (2017) Reactive Astrocytes Promote ALS-like Degeneration and Intracellular Protein Aggregation in Human Motor Neurons by Disrupting Autophagy through TGF-1. Stem Cell Reports 9:667-680.

Vidal PM, Lemmens E, Dooley D, Hendrix S (2013) The role of “ anti-inflammatory” cytokines in axon regeneration. Cytokine Growth Factor Rev 24:1-12.

Zhang K, Zhang Q, Deng J, Li J, Li J, Wen L, Ma J, Li C (2019) ALK5 signaling pathway mediates neurogenesis and functional recovery after cerebral ischemia/reperfusion in rats via Gadd45b. Cell Death Dis 10.

Zhang YE (2009) Non-Smad pathways in TGF-signaling. Cell Res 19:128-139.

## References

[B1] Abe K, Chu PJ, Ishihara A, Saito H (1996) Transforming growth factor-β 1 promotes re-elongation of injured axons of cultured rat hippocampal neurons. Brain Res 723:206–209. 10.1016/0006-8993(96)00253-3 8813400

[B2] Akhurst RJ, Hata A (2012) Targeting the TGF β signalling pathway in disease. Nat Rev Drug Discov 11:790–811. 23000686 10.1038/nrd3810 PMC3520610

[B3] Ata KA, Lennmyr F, Funa K, Olsson Y, Terént A (1999) Expression of transforming growth factor-beta1, 2, 3 isoforms and type I and II receptors in acute focal cerebral ischemia: an immunohistochemical study in rat after transient and permanent occlusion of middle cerebral artery. Acta Neuropathol 97:447–455. 10.1007/s004010051013 10334481

[B4] Bachmann LC, Lindau NT, Felder P, Schwab ME (2014) Sprouting of brainstem-spinal tracts in response to unilateral motor cortex stroke in mice. J Neurosci 34:3378–3389. 10.1523/JNEUROSCI.4384-13.2014 24573294 PMC6795311

[B5] Benowitz LI, Popovich PG (2011) Inflammation and axon regeneration. Curr Opin Neurol 24:577–583. 10.1097/WCO.0b013e32834c208d 21968547

[B6] Bryan B, Cai Y, Wrighton K, Wu G, Feng XH, Liu M (2005) Ubiquitination of RhoA by Smurf1 promotes neurite outgrowth. FEBS Lett 579:1015–1019. 10.1016/j.febslet.2004.12.074 15710384

[B7] Butler AM, Scotti Buzhardt ML, Erdogan E, Li S, Inman KS, Fields AP, Murray NR (2015) A small molecule inhibitor of atypical protein kinase C signaling inhibits pancreatic cancer cell transformed growth and invasion. Oncotarget 6:15297–15310. 10.18632/oncotarget.3812 25915428 PMC4558152

[B8] Cao Y, Wang C, Zhang X, Xing G, Lu K, Gu Y, He F, Zhang L (2014) Selective small molecule compounds increase BMP-2 responsiveness by inhibiting Smurf1-mediated Smad1/5 degradation. Sci Rep 4:4965. 10.1038/srep04965 24828823 PMC4021816

[B9] Carmichael ST, Kathirvelu B, Schweppe CA, Nie EH (2017) Molecular, cellular and functional events in axonal sprouting after stroke. Exp Neurol 287:384–394. 10.1016/j.expneurol.2016.02.007 26874223 PMC4980303

[B10] Cheng PL, Lu H, Shelly M, Gao H, Poo MM (2011) Phosphorylation of E3 ligase smurf1 switches its substrate preference in support of axon development. Neuron 69:231–243. 10.1016/j.neuron.2010.12.021 21262463

[B11] Cramer SC (2018) Treatments to promote neural repair after stroke. J Stroke 20:57–70. 10.5853/jos.2017.02796 29402069 PMC5836581

[B12] Curado F, Spuul P, Egaña I, Rottiers P, Daubon T, Veillat V, Duhamel P, Leclercq A, Gontier E, Génot E (2014) ALK5 and ALK1 play antagonistic roles in transforming growth factor-induced podosome formation in aortic endothelial cells. Mol Cell Biol 34:4389–4403. 10.1128/MCB.01026-14 25266657 PMC4248735

[B13] Do JL, Bonni A, Tuszynski MH (2013) SnoN facilitates axonal regeneration after spinal cord injury. PLoS One 8:e71906 10.1371/journal.pone.0071906 23936531 PMC3732222

[B14] Doyle KP, Cekanaviciute E, Mamer LE, Buckwalter MS (2010) TGFβ signaling in the brain increases with aging and signals to astrocytes and innate immune cells in the weeks after stroke. J Neuroinflammation 7:62. 10.1186/1742-2094-7-62 20937129 PMC2958905

[B15] Erdogan E, Lamark T, Stallings-Mann M, Jamieson L, Pellechia M, Aubrey Thompson E, Johansen T, Fields AP (2006) Aurothiomalate inhibits transformed growth by targeting the PB1 domain of protein kinase Ci. J Biol Chem 281:28450–28459. 10.1074/jbc.M606054200 16861740

[B16] Feltrin D, Pertz O (2012) Assessment of Rho GTPase signaling during neurite outgrowth. Methods Mol Biol 827:181–194. 22144276 10.1007/978-1-61779-442-1_13

[B17] Finelli MJ, Murphy KJ, Chen L, Zou H (2013) Differential phosphorylation of Smad1 integrates BMP and neurotrophin pathways through Erk/Dusp in axon development. Cell Rep 3:1592–1606. 10.1016/j.celrep.2013.04.011 23665221 PMC3677165

[B18] Goumans MJ, Valdimarsdottir G, Itoh S, Lebrin F, Larsson J, Mummery C, Karlsson S, ten Dijke P (2003) Activin receptor-like kinase (ALK) 1 is an antagonistic mediator of lateral TGFbeta/ALK5 signaling. Mol Cell 12:817–828. 10.1016/S1097-2765(03)00386-1 14580334

[B19] Gross CE, Bednar MM, Howard DB, Sporn MB (1993) Transforming growth factor-beta 1 reduces infarct size after experimental cerebral ischemia in a rabbit model. Stroke 24:558–562. 10.1161/01.STR.24.4.558 8465363

[B20] Hannila SS, Siddiq MM, Carmel JB, Hou J, Chaudhry N, Bradley PMJ, Hilaire M, Richman EL, Hart RP, Filbin MT (2013) Secretory leukocyte protease inhibitor reverses inhibition by CNS myelin, promotes regeneration in the optic nerve, and suppresses expression of the transforming growth factor-β signaling protein Smad2. J Neurosci 33:5138–5151. 10.1523/JNEUROSCI.5321-12.2013 23516280 PMC3684282

[B21] Ho TW, Bristol LA, Coccia C, Li Y, Milbrandt J, Johnson E, Jin L, Bar-Peled O, Griffin JW, Rothstein JD (2000) TGFbeta trophic factors differentially modulate motor axon outgrowth and protection from excitotoxicity. Exp Neurol 161:664–675. 10.1006/exnr.1999.7290 10686085

[B22] Jaskova K, Pavlovicova M, Cagalinec M, Lacinova L, Jurkovicova D (2014) TGFβ1 downregulates neurite outgrowth, expression of Ca2+ transporters, and mitochondrial dynamics of in vitro cerebellar granule cells. Neuroreport 25:340–346. 10.1097/WNR.0000000000000106 24535220

[B23] Jeon CY, Moon MY, Kim JH, Kim HJ, Kim JG, Li Y, Jin JK, Kim PH, Kim HC, Meier KE, Kim YS, Park JB (2012) Control of neurite outgrowth by RhoA inactivation. J Neurochem 120:684–698. 10.1111/j.1471-4159.2011.07564.x 22035369

[B24] Kaiser J, Maibach M, Salpeter I, Hagenbuch N, Souza VBC, Robinson MD, Schwab ME (2019) The spinal transcriptome after cortical stroke — in search of molecular factors regulating spontaneous recovery in the spinal cord. J Neurosci 39:2571–2518. 10.1523/JNEUROSCI.2571-18.2019 PMC6561692 30962276

[B25] Kang JS, Liu C, Derynck R (2009) New regulatory mechanisms of TGF-β receptor function. Trends Cell Biol 19:385–394. 10.1016/j.tcb.2009.05.008 19648010

[B26] Kennedy EJ, Scott JD (2015) Selective disruption of the AKAP signaling complexes. Methods Mol Biol 1294:137–150. 25783883 10.1007/978-1-4939-2537-7_11 PMC4722817

[B27] Knöferle J, Ramljak S, Koch JC, Tönges L, Asif AR, Michel U, Wouters FS, Heermann S, Krieglstein K, Zerr I, Bähr M, Lingor P (2010) TGF-β 1 enhances neurite outgrowth via regulation of proteasome function and EFABP. Neurobiol Dis 38:395–404. 10.1016/j.nbd.2010.02.011 20211260

[B28] Knuckey NW, Finch P, Palm DE, Primiano MJ, Johanson CE, Flanders KC, Thompson NL (1996) Differential neuronal and astrocytic expression of transforming growth factor beta isoforms in rat hippocampus following transient forebrain ischemia. Brain Res Mol Brain Res 40:1–14. 10.1016/0169-328x(96)00016-2 8840007

[B29] Kohta M, Kohmura E, Yamashita T (2009) Inhibition of TGF-beta1 promotes functional recovery after spinal cord injury. Neurosci Res 65:393–401. 10.1016/j.neures.2009.08.017 19744530

[B30] König HG, Kögel D, Rami A, Prehn JHM (2005) TGF-{beta}1 activates two distinct type I receptors in neurons: implications for neuronal NF-{kappa}B signaling. J Cell Biol 168:1077–1086. 10.1083/jcb.200407027 15781474 PMC2171851

[B31] Krakauer JW, Carmichael ST, Corbett D, Wittenberg GF (2012) Getting neurorehabilitation right: what can be learned from animal models? Neurorehabil Neural Repair 26:923–931. 10.1177/1545968312440745 22466792 PMC4554531

[B32] Lehrmann E, Kiefer R, Finsen B, Zimmer J, Hartung H-P (1995) Cytokines in cerebral ischemia: expression of transforming factor beta-1 (TGF-B1) mRNA in the postischemic adult rat hippocampus. Exp Neurol 131:114–123. 10.1016/0014-4886(95)90013-6 7895806

[B33] Li S, Nie EH, Yin Y, Benowitz LI, Tung S, Vinters HV, Bahjat FR, Stenzel-Poore MP, Kawaguchi R, Coppola G, Carmichael ST (2015) GDF10 is a signal for axonal sprouting and functional recovery after stroke. Nat Neurosci 18:1737–1745. 10.1038/nn.4146 26502261 PMC4790086

[B34] Li S, Gu X, Yi S (2017) The regulatory effects of transforming growth factor-β on nerve regeneration. Cell Transplant 26:381–394. 10.3727/096368916X693824 27983926 PMC5657701

[B35] Liddelow SA, Guttenplan KA, Clarke LE, Bennett FC, Bohlen CJ, Schirmer L, Bennett ML, Münch AE, Chung WS, Peterson TC, Wilton DK, Frouin A, Napier BA, Panicker N, Kumar M, Buckwalter MS, Rowitch DH, Dawson VL, Dawson TM, Stevens B, et al. (2017) Neurotoxic reactive astrocytes are induced by activated microglia. Nature 541:481–487. 10.1038/nature21029 28099414 PMC5404890

[B36] Maibach MA, Piovesana E, Kaiser J, Holm MM, Risic Z, Maurer M, Schwab ME (2020) Nogo-A beyond the RhoA/ROCK pathway – novel components of intracellular Nogo-A signaling cascades. bioRxiv. doi: https://doi.org/10.1101/2020.07.10.197368.

[B37] Makwana M, Jones LL, Cuthill D, Heuer H, Bohatschek M, Hristova M, Friedrichsen S, Ormsby I, Bueringer D, Koppius A, Bauer K, Doetschman T, Raivich G (2007) Endogenous transforming growth factor 1 suppresses inflammation and promotes survival in adult CNS. J Neurosci 27:11201–11213. 10.1523/JNEUROSCI.2255-07.2007 17942715 PMC6673043

[B38] Massagué J (2012) TGFβ signalling in context. Nat Rev Mol Cell Biol 13:616–630. 10.1038/nrm3434 22992590 PMC4027049

[B39] Metz GA, Whishaw IQ (2009) The ladder rung walking task: a scoring system and its practical application. J Vis Exp e1204. 10.3791/1204 PMC2796662 19525918

[B40] Morikawa M, Derynck R, Miyazono K (2016) TGF- β and the TGF-β family: context-dependent roles in cell and tissue physiology. Cold Spring Harb Perspect Biol 8:a021873 10.1101/cshperspect.a021873 27141051 PMC4852809

[B41] Murphy TH, Corbett D (2009) Plasticity during stroke recovery: from synapse to behaviour. Nat Rev Neurosci 10:861–872. 10.1038/nrn2735 19888284

[B42] Narimatsu M, Bose R, Pye M, Zhang L, Miller B, Ching P, Sakuma R, Luga V, Roncari L, Attisano L, Wrana JL (2009) Regulation of planar cell polarity by Smurf ubiquitin ligases. Cell 137:295–307. 10.1016/j.cell.2009.02.025 19379695

[B43] Niederöst B, Oertle T, Fritsche J, McKinney RA, Bandtlow CE (2002) Nogo-A and myelin-associated glycoprotein mediate neurite growth inhibition by antagonistic regulation of RhoA and Rac1. J Neurosci 22:10368–10376. 12451136 10.1523/JNEUROSCI.22-23-10368.2002 PMC6758757

[B44] Ozdamar B, Bose R, Barrios-Rodiles M, Wang H-R, Hang Y, Wrana JL (2005) Regulation of the polarity protein Par6 by TGF receptors controls epithelial cell plasticity. Science 307:1603–1609. 10.1126/science.1105718 15761148

[B45] Parikh P, Hao Y, Hosseinkhani M, Patil SB, Huntley GW, Tessier-Lavigne M, Zou H (2011) Regeneration of axons in injured spinal cord by activation of bone morphogenetic protein/Smad1 signaling pathway in adult neurons. Proc Natl Acad Sci USA 108:E99–E107. 10.1073/pnas.1100426108 21518886 PMC3093464

[B46] Prehn JHM, Backhauss C, Krieglstein J (1993) Transforming growth factor-beta1 prevents glutamate neurotoxicity in rat neocortical cultures and protects mouse neocortex from ischemic injury in vivo. J Cereb Blood Flow Metab 13:521–525. 10.1038/jcbfm.1993.67 8097519

[B47] Rønn LCB, Ralets I, Hartz BP, Bech M, Berezin A, Berezin V, Møller A, Bock E (2000) A simple procedure for quantification of neurite outgrowth based on stereological principles. J Neurosci Methods 100:25–32. 10.1016/S0165-0270(00)00228-4 11040363

[B48] Roome RB, Vanderluit JL (2015) Paw-dragging: a novel, sensitive analysis of the mouse cylinder test. J Vis Exp Advance online publication. Retrieved Apr 29, 2015. doi: 10.3791/52701. PMC4541598 25993447

[B49] Ruocco A, Nicole O, Docagne F, Ali C, Chazalviel L, Komesli S, Yablonsky F, Roussel S, MacKenzie ET, Vivien D, Buisson A (1999) A transforming growth factor-beta antagonist unmasks the neuroprotective role of this endogenous cytokine in excitotoxic and ischemic brain injury. J Cereb Blood Flow Metab Metab 19:1345–1353. 10.1097/00004647-199912000-00008 10598939

[B50] Sahai E, Garcia-Medina R, Pouysségur J, Vial E (2007) Smurf1 regulates tumor cell plasticity and motility through degradation of RhoA leading to localized inhibition of contractility. J Cell Biol 176:35–42. 10.1083/jcb.200605135 17190792 PMC2063621

[B51] Saijilafu, Hur E-M, Liu C-M, Jiao Z, Xu W-L, Zhou F-Q (2013) PI3K-GSK3 signalling regulates mammalian axon regeneration by inducing the expression of Smad1. Nat Commun 4:2690. 10.1038/ncomms3690 24162165 PMC3836055

[B52] Smith WB, Starck SR, Roberts RW, Schuman EM (2005) Dopaminergic stimulation of local protein synthesis enhances surface expression of GluR1 and synaptic transmission in hippocampal neurons. Neuron 45:765–779. 10.1016/j.neuron.2005.01.015 15748851

[B53] Stallings-Mann M, Jamieson L, Regala RP, Weems C, Murray NR, Fields AP (2006) A novel small-molecule inhibitor of protein kinase Ciota blocks transformed growth of non–small-cell lung cancer cells. Cancer Res 66:1767–1774. 10.1158/0008-5472.CAN-05-3405 16452237

[B54] Stegmüller J, Huynh MA, Yuan Z, Konishi Y, Bonni A (2008) TGF-Smad2 signaling regulates the Cdh1-APC/SnoN pathway of axonal morphogenesis. J Neurosci 28:1961–1969. 10.1523/JNEUROSCI.3061-07.2008 18287512 PMC6671436

[B55] Tadlock L, Yamagiwa Y, Hawker J, Marienfeld C, Patel T (2003) Transforming growth factor-β inhibition of proteasomal activity: a potential mechanism of growth arrest. Am J Physiol Cell Physiol 285:277–285. 10.1152/ajpcell.00550.2002 12646415

[B56] Thiede-Stan NK, Schwab ME (2015) Attractive and repulsive factors act through multi-subunit receptor complexes to regulate nerve fiber growth. J Cell Sci 128:2403–2412. 10.1242/jcs.165555 26116576

[B57] Tripathi P, Rodriguez-Muela N, Klim JR, de Boer AS, Agrawal S, Sandoe J, Lopes CS, Ogliari KS, Williams LA, Shear M, Rubin LL, Eggan K, Zhou Q (2017) Reactive astrocytes promote ALS-like degeneration and intracellular protein aggregation in human motor neurons by disrupting autophagy through TGF-β1. Stem Cell Reports 9:667–680. 10.1016/j.stemcr.2017.06.008 28712846 PMC5549875

[B58] Ueno M, Hayano Y, Nakagawa H, Yamashita T (2012) Intraspinal rewiring of the corticospinal tract requires target-derived brain-derived neurotrophic factor and compensates lost function after brain injury. Brain 135:1253–1267. 10.1093/brain/aws053 22436236

[B59] Vidal PM, Lemmens E, Dooley D, Hendrix S (2013) The role of “anti-inflammatory” cytokines in axon regeneration. Cytokine Growth Factor Rev 24:1–12. 10.1016/j.cytogfr.2012.08.008 22985997

[B60] Vohra BPS, Fu M, Heuckeroth RO (2007) Protein kinase C and glycogen synthase kinase-3 control neuronal polarity in developing rodent enteric neurons, whereas SMAD specific E3 ubiquitin protein ligase 1 promotes neurite growth but does not influence polarity. J Neurosci 27:9458–9468. 10.1523/JNEUROSCI.0870-07.2007 17728459 PMC2267823

[B61] Wahl AS, Schwab ME (2014) Finding an optimal rehabilitation paradigm after stroke: enhancing fiber growth and training of the brain at the right moment. Front Hum Neurosci 8:1–13. 25018717 10.3389/fnhum.2014.00381 PMC4072965

[B62] Walshe TE, Leach LL, D'Amore PA (2011) TGF-β signaling is required for maintenance of retinal ganglion cell differentiation and survival. Neuroscience 189:123–131. 10.1016/j.neuroscience.2011.05.020 21664439 PMC3150228

[B63] Wang HR, Zhang Y, Ozdamar B, Ogunjimi AA, Alexandrova E, Thomsen GH, Wrana JL (2003) Regulation of cell polarity and protrusion formation by targeting RhoA for degradation. Science 302:1775–1779. 10.1126/science.1090772 14657501

[B64] Wang L, Zhu Y, Sharma K (1998) Transforming growth factor-beta1 stimulates protein kinase A in mesangial cells. J Biol Chem Chem 273:8522–8527. 10.1074/jbc.273.14.8522 9525967

[B65] Watson BD, Dietrich WD, Busto R, Wachtel MS, Ginsberg MD (1985) Induction of reproducible brain idarction by photochemically initiated thrombosis. Ann Neurol 17:497–504. 10.1002/ana.410170513 4004172

[B66] Weiss A, Attisano L (2013) The TGFbeta superfamily signaling pathway. Wiley Interdiscip Rev Dev Biol 2:47–63. 10.1002/wdev.86 23799630

[B67] Yang H, Li G, Wu JJ, Wang L, Uhler M, Simeone DM (2013) Protein kinase a modulates transforming growth factor-β signaling through a direct interaction with Smad4 protein. J Biol Chem 288:8737–8749. 10.1074/jbc.M113.455675 23362281 PMC3605691

[B68] Yi JJ, Barnes AP, Hand R, Polleux F, Ehlers MD (2010) TGF-β signaling specifies axons during brain development. Cell 142:144–157. 10.1016/j.cell.2010.06.010 20603020 PMC2933408

[B69] Zeiler SR, Krakauer JW (2013) The interaction between training and plasticity in the poststroke brain. Curr Opin Neurol 26:609–616. 10.1097/WCO.0000000000000025 24136129 PMC4012223

[B70] Zhang J, Li Z, Chen F, Liu H, Wang H, Li X, Liu X, Wang J, Zheng Z (2017) TGF-β1 suppresses CCL3/4 expression through the ERK signaling pathway and inhibits intervertebral disc degeneration and inflammation-related pain in a rat model. Exp Mol Med 49:e379. 10.1038/emm.2017.136 28935976 PMC5628275

[B71] Zhang K, Zhang Q, Deng J, Li J, Li J, Wen L, Ma J, Li C (2019) ALK5 signaling pathway mediates neurogenesis and functional recovery after cerebral ischemia/reperfusion in rats via Gadd45b. Cell Death Dis 10:360 10.1038/s41419-019-1596-z 31043581 PMC6494915

[B72] Zhang YE (2009) Non-Smad pathways in TGF-β signaling. Cell Res 19:128–139. 10.1038/cr.2008.328 19114990 PMC2635127

[B73] Zhu Y, Culmsee C, Roth-Eichhorn S, Krieglstein J (2001) Beta(2)-adrenoceptor stimulation enhances latent transforming growth factor-beta-binding protein-1 and transforming growth factor-beta1 expression in rat hippocampus after transient forebrain ischemia. Neuroscience 107:593–602. 10.1016/s0306-4522(01)00357-8 11720783

[B74] Zou H, Ho C, Wong K, Tessier-Lavigne M (2009) Axotomy-induced Smad1 activation promotes axonal growth in adult sensory neurons. J Neurosci 29:7116–7123. 10.1523/JNEUROSCI.5397-08.2009 19494134 PMC2739099

